# The impact of recreational cannabinoid legalization on utilization in a pregnant population

**DOI:** 10.3389/fpubh.2024.1278834

**Published:** 2024-02-20

**Authors:** Jacob Torres, Colton Miller, Michael Apostol, Jessica Gross, Jessie R. Maxwell

**Affiliations:** ^1^School of Medicine, University of New Mexico, Albuquerque, NM, United States; ^2^Clinical and Translational Science Center, University of New Mexico, Albuquerque, NM, United States; ^3^Department of Pediatrics, University of New Mexico, Albuquerque, NM, United States; ^4^Department of Neurosciences, University of New Mexico, Albuquerque, NM, United States

**Keywords:** prenatal cannabinoid exposure, prenatal cannabis, pregnancy, pregnant substance users, tetrahydrocannabinol

## Abstract

**Background:**

Marijuana potency and utilization both continue to increase across the United States. While the overall prevalence of cannabinoid utilization during pregnancy has been surveyed in various studies, the direct impact of changing governmental policies on pregnancy use is less characterized. Thus, we aimed to investigate how the legalization of recreational cannabinoid products impacted use during pregnancy in the state of New Mexico.

**Methods:**

Participants who had a live birth during two study epochs were included: pre-legalization (Epoch 1: 1 January 2019–31 March 2021) and post-legalization (Epoch 2: 1 November 2021–30 November 2022). Participants were further divided into case group [prenatal cannabinoid exposure (PCE)] vs. control (no PCE), with cases being identified by documented self-report or a positive laboratory toxicology test for cannabinoid use during pregnancy.

**Results:**

A total of 1,191 maternal/infant dyads were included in Epoch 1, and 378 maternal/infant dyads were included in Epoch 2. In Epoch 1, 788 dyads were controls with 403 cases, while Epoch 2 had 292 controls and 86 cases. Interestingly there was a significant decrease in self-report or positive laboratory toxicology tests in Epoch 2 compared to Epoch 1. Infants born following PCE in both Epoch groups were more commonly born via Cesarean section, had significantly smaller birth weight, length, and head circumference as well as significantly lower Apgar scores at 1 and 5 min.

**Conclusion:**

The finding of decreased reported cannabinoid use in the post-legalization group is contradictory to previous studies which have shown increased rates of cannabinoid use after legalization. This could be due to multiple factors including changes in screening practices, the COVID-19 pandemic, and lack of commercialization of THC products. Additional studies are needed to further characterize how changing governmental policies impacts utilization during pregnancy.

## Introduction

Marijuana (*Cannabis sativa*) is a plant native to Eastern Asia and contains over 100 unique cannabinoids responsible for its psychoactive and medicinal properties (1). Of the more than 100 cannabinoids, trans-delta-9-tetrahydrocannabinol (THC) is the most psychoactive (2, 3). As a lipophilic molecule, THC can readily cross the placenta and penetrate the fetal central nervous system (CNS) (4–9). The concentration of THC in cannabinoid products used in the present day is up to 300% greater than the 1990s, with nearly triple the potency (10, 11). Compounding these changes is the increasing availability of cannabinoid products as states across the country continue to legalize them for recreational use.

**Figure 1:**
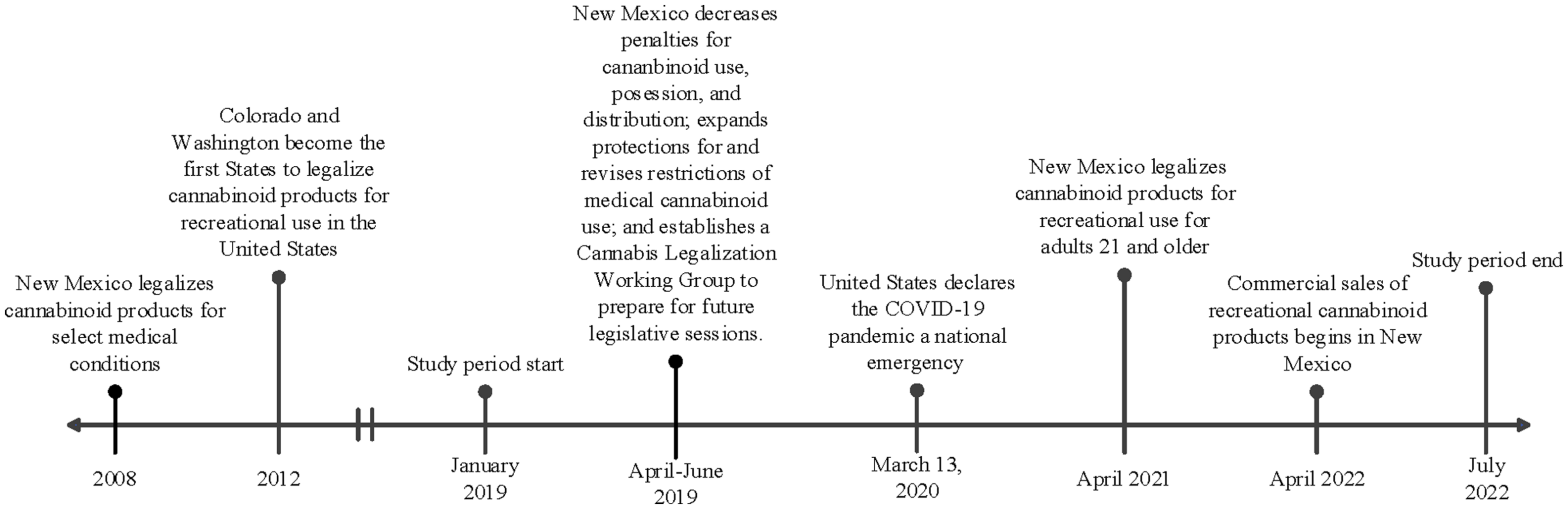
Timeline of key regulatory and contextual events in New Mexico and United States in relation to the study period.

In April 2021, the state of New Mexico passed the Cannabis Regulation Act, legalizing the consumption, purchase, possession, and cultivation of marijuana or other products containing THC (12). This came shortly after the state decriminalized possession of these products in 2019 (see Figure 1 for a timeline of key regulatory events in New Mexico and the United States). To date, 22 other states have legalized cannabinoid products for recreational use (13). Many of these states have commercial markets for cannabinoid products, and in New Mexico, licensed retail sales began in April 2022 (14). However, marijuana is currently classified as a schedule I drug by the United States Drug Enforcement Administration (DEA) and is an illegal drug federally. Despite the conflicting legalization status at the state and national level, cannabinoids remain the most widely used illicit drug in the United States. This also translates to pregnancy, where cannabinoid products remain the most used illicit substance in pregnancy (10). The impacts of legalizing cannabinoid products have been a controversial and pressing public health concern, and many studies show an increase in utilization after legalization (15–17). One study in particular analyzed data from six states that legalized cannabinoid products and the analysis revealed an increase in utilization in pregnant individuals throughout the preconception, prenatal, and postpartum stages (16). Legalization has also been shown to normalize use and minimize perceived harms. Dispensaries in Colorado recommend cannabinoid products for the treatment of morning sickness in pregnancy (18). Approximately 70% of pregnant and non-pregnant individuals believe there is only slight or no risk of harm from using cannabinoids during pregnancy (10, 19).

Confounding the notion that cannabinoids are safe is the difficulty obtaining high quality studies to determine the impact on fetal growth and development. The concomitant use of other substances such as tobacco or alcohol, in the setting of changing cannabinoid potency, creates difficulty ascertaining if changes in fetal growth and development are directly related to the consumption of cannabinoids during pregnancy (20–22). Increased stress from social and environmental stressors, including socioeconomic status and access to prenatal care also directly impact neonatal outcomes (23–26). Additionally, THC itself may have adverse impacts on maternal stress and anxiety throughout pregnancy (27, 28). THC is a partial agonist of the cannabinoid receptor 1 (CB_1_) (27–29). While CB_1_ receptor activation can have an anxiolytic effect at low doses, it has a biphasic effect with higher doses of THC having an anxiogenic effect (27). Through this biphasic effect, the increased potency and concentration of THC products may lead to increased levels of maternal stress and anxiety, which may also impact fetal well-being (23).

Despite these challenges, it is known that cannabinoid receptors are present throughout the fetus during development, including in the CNS, cardiovascular, respiratory, immune, reproductive, hepatic, muscular, gastrointestinal, and skeletal systems, implicating the potential for vast alterations in development (20, 30–33). The expression of the primary brain cannabinoid receptor (CB_1_) is highest during gestation and is related to the development of other neurotransmitters such as the opioid and dopaminergic systems (1, 30, 34). It is also intensely expressed in the mesocorticolimbic system (1, 34–37). Cannabinoids and their receptors play a prominent role in synaptogenesis, neurite formation, neural migration, proliferation, and maturation (35, 36, 38–40). Thus, it is not surprising that several studies have observed alterations in dopaminergic activity in the amygdala, alterations in memory, verbal reasoning, visual–spatial processing, attention, sleep efficiency, increased impulsivity, and hyperactivity later in childhood following prenatal cannabinoid exposure (1, 32–34, 41–45). A review of the overall impact of cannabinoid exposure on an individual can be found in Lin et al. (46). Our understanding of population and developmental outcomes following *in-utero* cannabinoid exposure is evolving. Additional studies are needed to better characterize the consequences of prenatal cannabinoid exposure.

Due to the clinical implications of use following legalization, we sought to determine how legalization of cannabinoids would impact utilization in a pregnant population in New Mexico. We hypothesized that following legalization, a rise in the observed cannabinoid use rates would occur in the pregnant population.

## Materials and methods

Following approval by the University of New Mexico Health Sciences Center Institutional Review Board (IRB), study participants were identified through use of an honest broker. Individuals were included if they delivered a liveborn infant during the two epochs (pre-legalization and post-legalization) at the University of New Mexico Hospital (UNMH). UNMH serves the immediate urban and surrounding rural areas of New Mexico and accepts transports from across the state and surrounding states. The first epoch (pre-legalization) included individuals that delivered between 1 January 2019 and 31 March 2021. The second epoch (post-legalization) included individuals that delivered between 1 November 2021 and 30 November 2022. Individuals who delivered in between the two epochs were excluded from the study, as this was a transition period around the legalization of recreational cannabinoid use. The timeline of key regulatory and contextual events in New Mexico are shown in Figure 1, including the study periods.

The two epoch groups were further divided into prenatal cannabinoid exposure (case group, hereafter referred to as PCE) and no known prenatal cannabinoid exposure (control group). Participants were included in the case group if there was self-reported cannabinoid use documented during the pregnancy or if at least one laboratory test was positive for metabolites of marijuana during the pregnancy. Individuals with no positive laboratory and no self-reported use were included in the control group. No other laboratory tests were analyzed as part of the study protocol.

Information related to substance use in pregnancy, demographic information, and birth outcomes were obtained for all participants from the medical record. Specifically, maternal demographic information included maternal age, marital status (categorized as single/separated/divorced, married/civil union, partnered, minor/other), ethnicity (categorized as Hispanic/Latino, Non-Hispanic/Latino, other), race (categorized as White, Black/African American, American Indian/Alaska Native, Asian/Pacific Islander, other), and medical insurance type (categorized as Medicaid, commercial/self-pay, other/other government). Maternal information during pregnancy was obtained including gravida, parity, trimester of initiation of prenatal care, number of prenatal care visits, illicit substance use other than cannabinoids, chronic and perinatal maternal medical conditions, and prenatal medications. Infant information obtained included the sex, gestational age at birth, mode of delivery (vaginal or cesarean section), Apgar scores at 1 and 5 min, birth weight, birth length, head circumference, birth growth assessment (small, appropriate, or large for gestational age), congenital abnormalities if present, and need for oxygen therapy at birth.

Demographic and clinical characteristics were summarized using percentages for categorical variables and means and standard deviations for continuous variables. Statistical analysis was completed along two primary lines of comparison. First, analysis was done comparing demographic and clinical characteristics in maternal/infant dyads in total controls vs. total cases from all epochs combined. Second, analysis was completed comparing these same characteristics in maternal/infant dyad groups (control vs. case) in Epoch 1 vs. Epoch 2. Statistical analysis was completed primarily through two-sample t-test assuming equal variances for continuous variables and chi-square tests for categorical variables, with expected values for cannabinoid utilization calculated within the chi-square test. Statistical significance was defined with an alpha level of 0.05.

Following these analyses, we performed three multiple logistic regression models to ascertain the independent effects of COVID-19 shutdown, insurance type, other substance use, and legalization on the likelihood of prenatal cannabinoid exposure. In all models, the period of COVID-19 shutdown was determined by the New Mexico Department of Health guidelines; the shutdown officially began on 11 March 2020, and ended on 31 March 2022. In Model 1, the COVID-19 shutdown was coded as a binary variable. All participants in Epoch 1 with infant birthdates prior to 11 March 2020 were classified into the non-COVID-19 shutdown group, and those with infant birthdates after 11 March 2020 were classified into the COVID-19-shutdown group. All Epoch 2 participants with infant birthdates prior to 31 March 2022 were classified into the COVID-19 shutdown group, and all participants with infants born after that date were classified into the non-COVID-19-shutdown group.

Model 2 separated the data into three COVID-19 periods: (1) pre-COVID-19 shutdown (infant birthdates prior to 11 March 2020), (2) during COVID-19 shutdown (infant birthdates between 11 March 2020 and 31 March 2022), and (3) post-COVID-19 shutdown (infant birthdates after 31 March 2022). Model 3 separated the data into three time periods according to cannabinoid accessibility: (1) pre-legalization (infant birthdates prior to 1 April 2021), (2) post-legalization/pre-legal retail sales (infant birthdates between 1 April 2021 and 1 April 2022), and (3) post-legal retail sales (infant birthdates after 1 April 2022).

We used odds ratios to quantify the strength and direction of associations and assessed the overall fit of all models using McFadden’s pseudo-*R* (2). A significance threshold of 0.05 was used to determine the statistical significance of the model and individual predictors.

## Results

**Figure 2:**
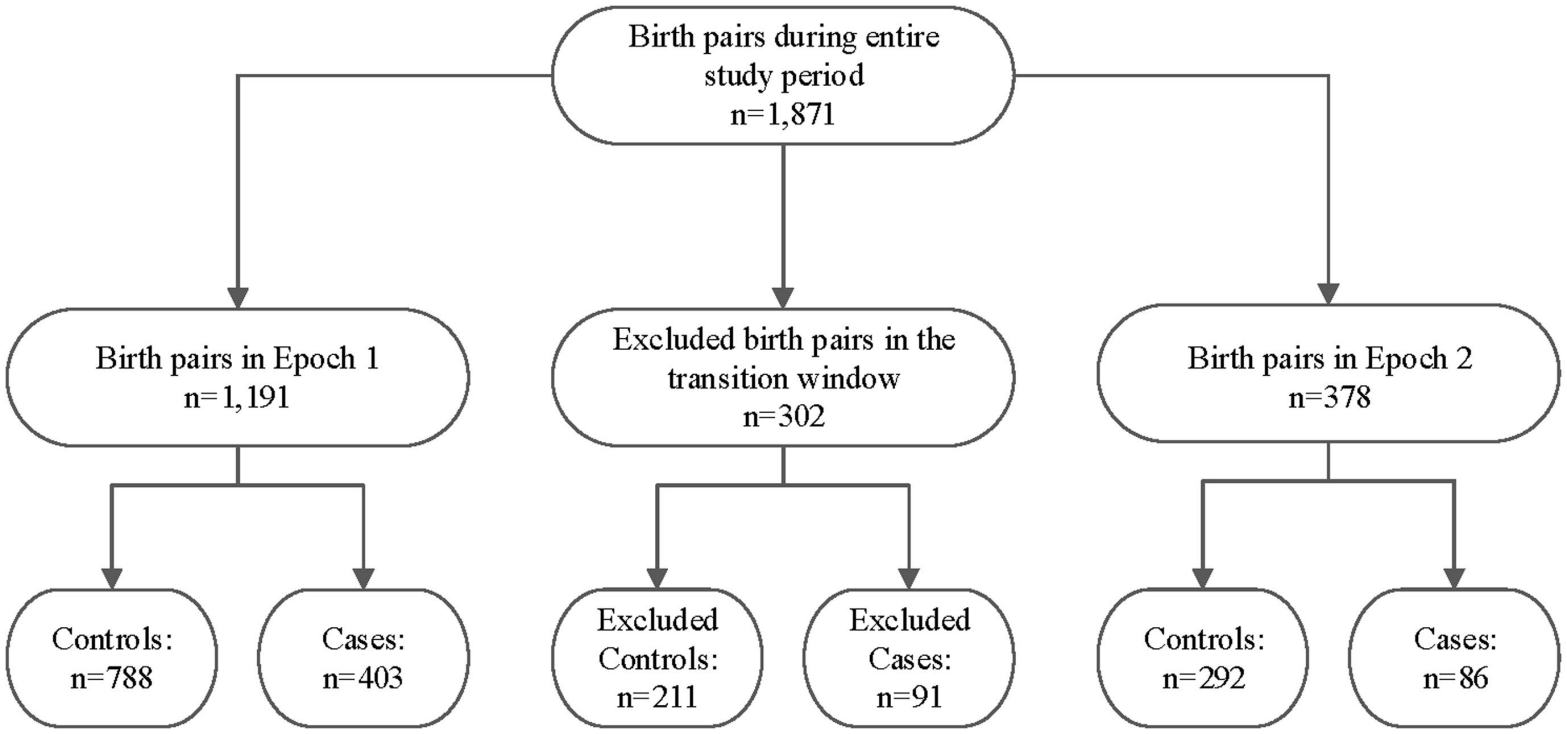
Study sample breakdown by epoch, transition window, and case/control status. Epoch 1: The pre-legalization window of the study period (1 January 2019–31 March 2021) Transition window: The period between when the New Mexico Cannabis Regulation Act was signed and several months after it went into effect (1 April–31 October 2021) Epoch 2: The post-legalization window of the study period (1 November 2021–November 2022).

A total of 1,871 maternal/infant dyads were admitted to UNMH during the entire study duration, as shown in Figure 2. However, 302 of the infants were born during the transition period between pre- and post-legalization and were excluded from further analysis. The pre-legalization Epoch (Epoch 1) included 1,191 maternal/infant dyad, of which 788 were controls (no known prenatal cannabinoid use) and 403 were cases (confirmed prenatal cannabinoid use). The post-legalization Epoch (Epoch 2) included 378 maternal/infant dyads, with 292 controls and 86 cases.

There was no difference in maternal age between Epoch 1 and Epoch 2, as well as between all controls compared to all PCE (*p* = 0.07, see Table 1). Interestingly, there was a difference in maternal marital status, with more individuals reporting single status in Epoch 1 compared to Epoch 2 (*p* < 0.05), as well as all controls compared to all PCE (*p* < 0.001). No statistical difference was noted in maternal ethnicity between Epoch 1 and Epoch 2, but fewer individuals identified as Asian/Pacific Islander in PCE compared to the control group (*p* < 0.001, see Table 1). More individuals had Medicaid insurance in the PCE group than expected (*p* < 0.001, see Table 1). Fewer individuals had adequate prenatal care (defined as <3 prenatal visits during the pregnancy) in Epoch 2 compared to Epoch 1 (*p* < 0.01), with a higher number of individuals in the PCE group having no or inadequate prenatal care (*p* < 0.001, see Table 1).

**Table 1:** Characteristics of the maternal and infant study participants stratified by case and control status.

Variable		All controls (*N =* 1,080)		All cases (*N =* 489)		Value of *p*
**Maternal characteristics**							
Age in years (Mean ± SD)			29.3 ± 5.5		29.3 ± 5.5		0.071^1^
Marital status				<0.001^2*^
Single/Separated/Divorced	351 (32.5%)		259 (53.0%)		
Married/Civil Union	458 (42.4%)		72 (14.7%)		
Partnered	247 (22.9%)		150 (30.7%)		
Minor/Other	24 (2.2%)		8 (1.6%)	
Ethnicity				0.342^2^
Hispanic/Latino	622 (57.6%)		296 (60.5%)		
Not Hispanic/Latino	433 (40.1%)		186 (38.0%)		
Other	25 (2.3%)		7 (1.4%)		
Race				<0.001^2*^
White	772 (71.5%)		360 (73.6%)		
Black/African American	34 (3.1%)		38 (7.8%)		
American Indian, Alaska Native	165 (15.3%)		65 (13.3%)		
Asian/Pacific Islander	57 (5.3%)		3 (0.6%)		
Other	52 (4.8%)		23 (4.7%)		
Medical insurance type					<0.001^2*^
Medicaid	435 (40.3%)		391 (80.0)		
Commercial/self-pay	462 (42.8)		59 (12.1%)		
Other/Other government	183 (16.9%)		39 (8.0%)		
Prenatal care				<0.001^2*^
Adequate	921 (85.3%)		350 (71.6%)		
None/Not Adequate	72 (6.7%)		91 (18.6%)		
Unknown	87 (8.0%)		47 (9.6%)		
**Infant characteristics**							
Sex: female		519 (48.1%)		243 (49.7%)		0.548^2^
Race				<0.001^2*^
White	781 (72.3%)		355 (72.6%)		
Black/African American	44 (4.1%)		47 (9.6%)		
American Indian, Alaska Native	172 (15.9%)		71 (14.5%)		
Asian/Pacific Islander	51 (4.7%)		1 (0.2%)		
Other	32 (3.0%)		15 (3.1%)		
Ethnicity				0.157^2^
Hispanic/Latino	634 (58.7%)		296 (60.5%)		
Not Hispanic/Latino	407 (37.7%)		167 (34.2%)		
Other	39 (3.6%)		26 (5.3%)		

^1^Based on pooled variances *t*-test; ^2^Based on Chi-Squared analysis; ^*^Statistically significant; SD, Standard Deviation.

Utilization of substances other than cannabinoids was different between groups as well (see Table 2). Tobacco use was significantly different between Epoch 1 and Epoch 2 (*p* < 0.05), and more individuals in all cases used tobacco compared to individuals with no prenatal cannabinoid exposure (*p* < 0.001, see Table 2). Similarly, alcohol use was higher in the PCE group compared to those in the control group (*p* < 0.001, see Table 2), although no difference was noted between Epoch 1 and Epoch 2. Opioid use was also significantly higher in the PCE group compared to the controls (*p* < 0.001, see Table 2).

**Table 2:** Substance use in addition to marijuana in all controls and cases.

Variable	All controls (*N =* 1,080)	All cases (*N =* 489)	Value of *p*
Tobacco/Nicotine	45	225	<0.001
Alcohol	16	48	<0.001
Opioid (including prescription)	52	199	<0.001

**Figure 3:**
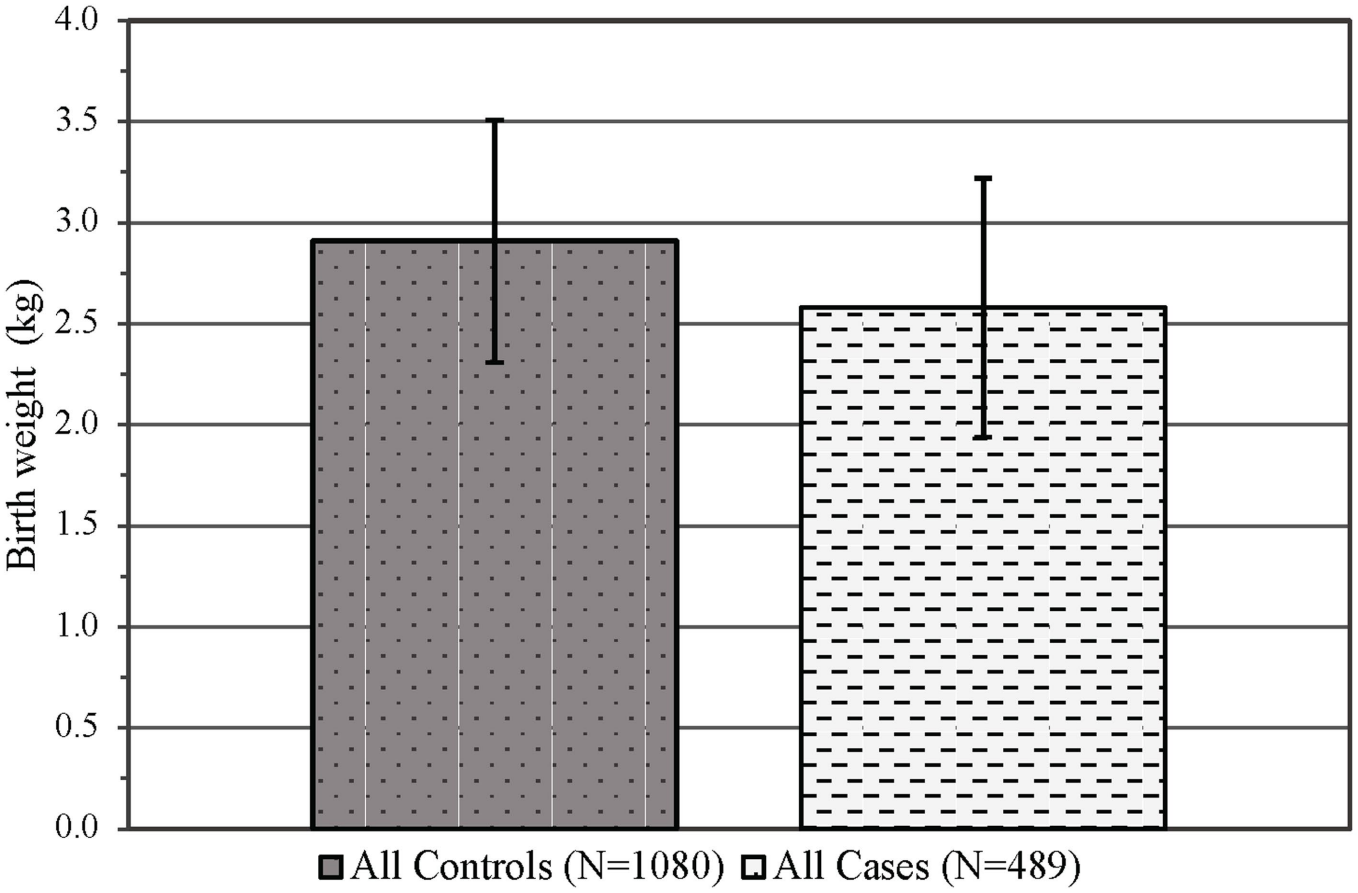
Infant mean birth weight in kilograms (±SD) by control and case status across both epochs. *p* < 0.001, SD, Standard deviation.

There was no difference observed in the infant sex at birth between groups (*p* = 0.55, see Table 1), although the gestational age was significantly decreased in the PCE group compared to the controls (*p* < 0.01, see Table 3). Similar to maternal results, there were fewer individuals identifying as Asian/Pacific Islander in the PCE group compared to the controls (*p* < 0.001, see Table 1). In the control group, more deliveries occurred via vaginal delivery than expected, while more of the PCE group was delivered via Cesarean section (*p* < 0.05, see Table 3). Infants born following prenatal cannabinoid exposure were significantly lower weight (*p* < 0.001, see Figure 3), shorter in length (*p* < 0.001, see Table 3), and had smaller head circumferences (*p* < 0.001, see Table 3) at birth compared to infants in the control group. Additionally, the Apgar scores at 1 and 5 min were significantly lower in the PCE group compared to the control group (*p* < 0.001 and *p* < 0.01, respectively, see Table 3).

**Table 3:** Infant birth characteristics stratified by case and control status.

Variable		All controls (*N =* 1,080)	All cases (*N =* 489)	*p-*value
Gestational age at birth (Mean ± SD)		38.50 ± 2.25	38.11 ± 2.74	<0.01
Mode of delivery				<0.05
Vaginal birth	790 (73.2%)	329 (67.3%)	
Cesarean section	290 (26.9%)	160 (32.7%)	
Apgar scores (Mean ± SD)	
1 min	7.48 ± 1.57	7.17 ± 1.77	<0.001
5 min	8.73 ± 0.90	8.56 ± 1.18	<0.01
Birth length (Mean ± SD)		49.01 ± 4.56	47.07 ± 4.86	<0.001
Birth FOC (Mean ± SD)		33.42 ± 2.72	32.26 ± 3.20	<0.001

SD, Standard deviation; FOC, Frontal-occipital circumference.

**Figure 4:**
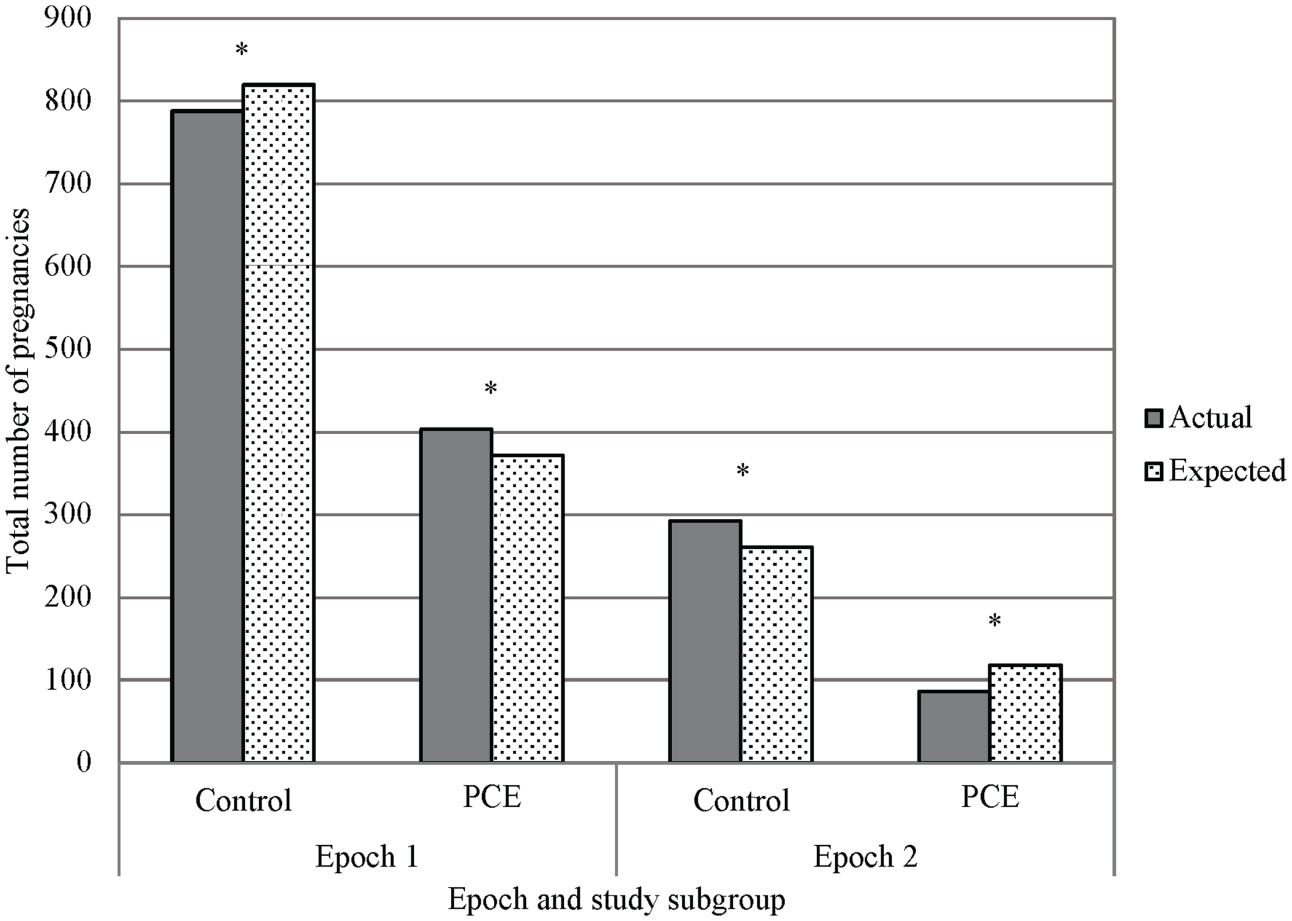
Primary outcome, marijuana exposure vs. control in epochs 1 and 2. A chi-squared analysis comparing PCE and no PCE in pregnancy before and after THC was legalized (Epochs 1 and 2 respectively) showed less PCE in Epoch 2 than what would be expected, and PCE in Epoch 1 was greater than what would be expected. Additionally, there was a noted decrease in reported PCE in Epoch 2 when compared to Epoch 1. ^*^p < 0.001; PCE, Prenatal cannabis exposure.

The utilization of cannabinoids during pregnancy was significantly different between Epoch 1 and Epoch 2. Interestingly, the utilization prior to legalization (Epoch 1) was higher than expected and was lower than expected following legalization (Epoch 2, *p* < 0.001, see Figure 4). In Epoch 2, the number of individuals identified with cannabinoid use during pregnancy through verbal screen alone was significantly higher than expected, with the number of individuals identified through toxicology screen alone or toxicology screen and verbal report both significantly lower than expected (*p* < 0.001). The number of individuals with no cannabinoid use was lower than expected prior to legalization (Epoch 1) and was higher than expected following legalization (Epoch 2, *p* < 0.001, see Figure 4).

### Model 1

The logistic regression model was statistically significant, χ2(7) = 594.02, *p* < 0.001. The model explained 31.0% (McFadden pseudo-*R*^2^) of the variance in PCE use and correctly classified 81.5% of patients in the PCE group (see Table 4). Most independent variables included in the model significantly contributed to variation in cannabinoid use during pregnancy, however the difference between participants in the commercial/self-pay and other/other government insurance groups was not significant (*p* = 0.068), and the COVID-19 shutdown did not contribute significantly to the variation in cannabinoid use among participants (*p* = 0.072).

**Table 4:** Model 1 (binary COVID-19 shutdown variable) classification table.

Classification table	Predicted		% correct
Observed	non-PCE	PCE	
non-PCE	1,019	61	94.35
PCE	229	260	53.17
Overall % Correct			81.52

The results of the logistic regression are shown in Table 5. Participants in the pre-legalization epoch were nearly twice as likely (OR = 1.95) to use cannabinoids than post-legalization epoch participants, which is consistent with the results of the chi-square tests. Utilization of substances other than cannabinoids contributed significantly to the variation in cannabinoid use among participants. Participants who used tobacco were approximately 10.8 times more likely to be in the PCE group compared to those who did not use tobacco. Similarly, participants who used alcohol were 5.4 times more likely to be in the PCE group than those who did not use alcohol, and participants who used opiates were 3.9 times more likely to be in the PCE group than those who did not use opiates. Insurance type and epoch also had significant independent effects on PCE status. Participants with Medicaid were 4.6 more likely to be in the PCE group relative to participants in the commercial/self-pay insurance group.

**Table 5:** Model 1 (binary COVID-19 shutdown variable) results.

	Results					Estimate	Standard error	Odds ratio	z	*p*
Intercept	−3.11	0.23	0.04	−13.60	0.00
Tobacco use (yes)	2.38	0.19	10.79	12.32	0.00
Alcohol use (yes)	1.68	0.36	5.37	4.65	0.00
Opiate use (yes)	1.35	0.23	3.86	6.00	0.00
COVID-19 shutdown (yes)	0.25	0.14	1.28	1.80	0.07
Insurance (Medicaid)	1.53	0.17	4.61	8.78	0.00
Insurance (other/other government)	0.45	0.25	1.57	1.83	0.07
Epoch (pre-legalization)	0.67	0.17	1.95	3.87	0.00

### Model 2

To further examine the decrease in utilization in Epoch 2, we tested two additional models. Model 2 separated the dataset into three time periods relating to the COVID-19 shutdown. We hypothesized that the reduction in utilization identified in the chi-square analyses stemmed from variation in utilization rates before, during, and after the shutdown. Additionally, any potential increase after the shutdown might not have been detectable in the initial analyses. However, the results confirm COVID-19 shutdown did not have a significant effect on cannabinoid utilization in our sample. Therefore, this model did not further explain the decrease in utilization following legalization (see Table 6).

**Table 6:** Model 2 results (3-phase COVID-19 shutdown variable).

	Estimate	Standard error	Odds ratio	z	*p*
(Intercept)	−2.93	0.29	0.05	−10.30	0.00
Tobacco use (yes)	2.38	0.19	10.78	12.30	0.00
Alcohol use (yes)	1.69	0.36	5.39	4.66	0.00
Opiate use (yes)	1.37	0.23	3.95	6.05	0.00
Insurance (Medicaid)	1.53	0.17	4.62	8.79	0.00
Insurance (other/other government)	0.45	0.25	1.57	1.82	0.07
COVID-19 shutdown (during)	0.18	0.15	1.20	1.17	0.24
COVID-19 shutdown (after)	−0.38	0.35	0.68	−1.08	0.28
Epoch (pre-legalization)	0.51	0.22	1.67	2.31	0.02

### Model 3

Model 3 divided the dataset into three time periods relating to the availability of cannabinoids: (1) pre-retail availability, and (2) post-retail availability. Our hypothesis was that cannabinoid utilization was not more readily accessible until licensed retail stores opened. Since most participants in Epoch 2 predated the opening of licensed retail stores this may have prevented us from detecting an increase associated with legalization. However, we did not find support for this hypothesis; participants in the pre-retail group were 2.44 times more likely to utilize cannabinoids than those in the post-retail group (Table 7).

**Table 7:** Model 3 (availability variable) results.

	Estimate	Standard error	Odds ratio	z	*p*
(Intercept)	−2.41	0.17	0.09	−13.95	0.00
Tobacco use (yes)	2.38	0.19	10.83	12.35	0.00
Alcohol use (yes)	1.66	0.36	5.24	4.62	0.00
Opiate use (yes)	1.39	0.23	4.00	6.11	0.00
COVID-19 shutdown (yes)	0.03	0.14	1.03	0.23	0.82
Insurance (Medicaid)	1.53	0.17	4.60	8.78	0.00
Insurance (other/other government)	0.45	0.25	1.56	1.81	0.07
Availability (post-legal/post-retail)	−0.90	0.28	0.41	−3.26	0.00

## Discussion

The results of this study did not support our hypothesis that cannabinoid use would increase during the post-legalization period; rather, there was a statistically significant decrease in observed utilization. This is inconsistent with other studies which have found increased rates of cannabinoid use in both the general population (15, 47, 48) and specifically during the pre-conception, prenatal, and post-partum time periods (16, 17) following the legalization period in other states. This trend of increased use of cannabinoid products during pregnancy has also been shown in other countries including Canada (17).

**Figure 5:**
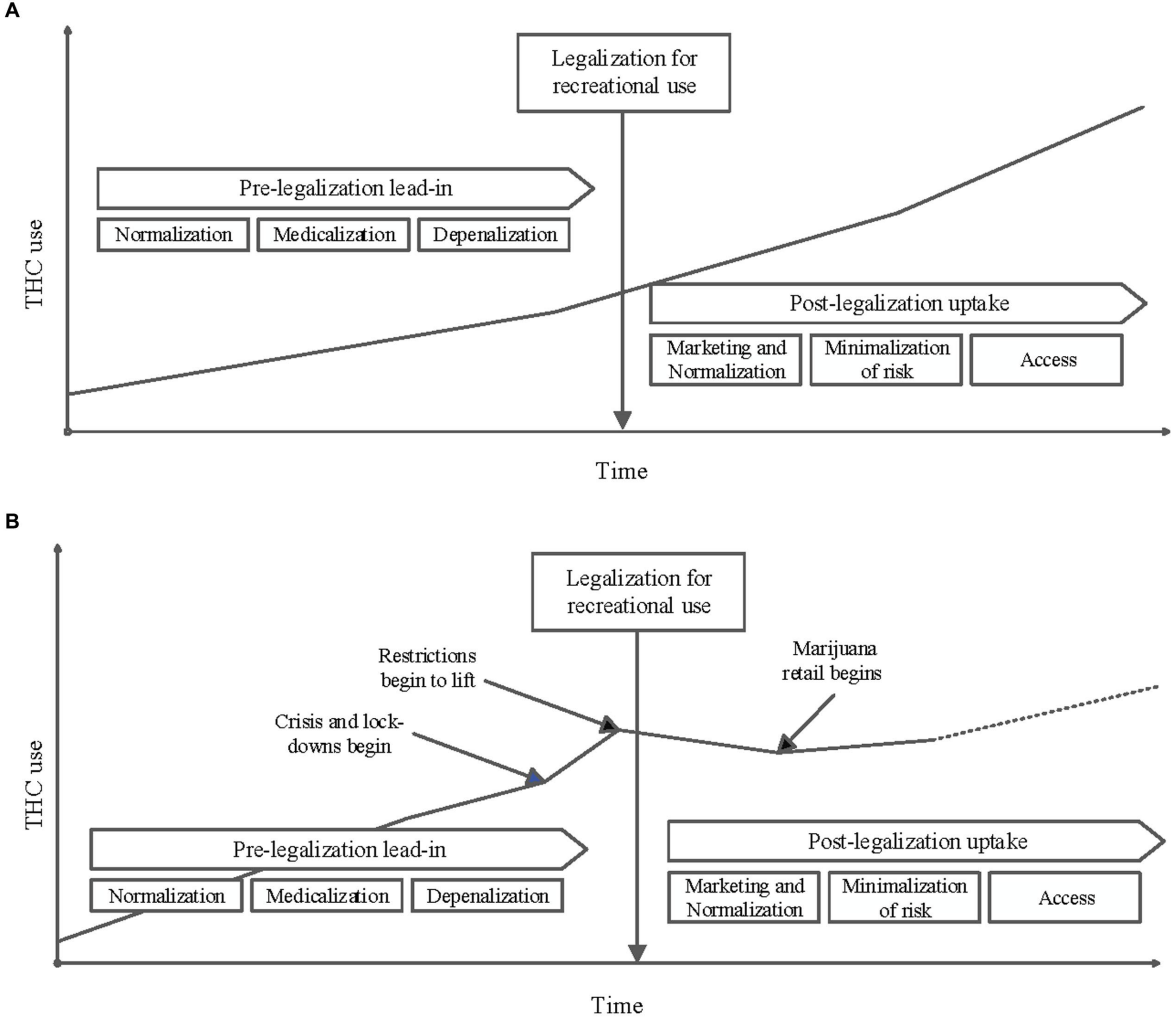
**(A)** Conceptual diagram of THC use in the population over time before and after it is legalized for recreational use: An increase in marijuana consumption is often observed in the time leading up to its legalization for recreational use. This is often attributed to changing social norms, decriminalization and depenalization, and legalizing marijuana for medicinal use. A further increase in use in the population is frequently seen post-legalization. This increase if often attributed to increased access that comes with commercial dispensaries, marketing, minimalization of risks associated with use, continued normalization and further medicalization. **(B)** Conceptual diagram of THC use in the study population over time as influenced by key events: The results of this study do not follow the trends depicted in A. While a pre-legalization lead-in period is visualized above, it is possible that the stresses of the Pandemic caused a sharp increase in use before legalization. As restrictions lifted in the Pandemic response, it is possible that this relative increase in THC use have started to decrease. The observed decrease in THC use after legalization also could be a reflection of decreased reporting secondary to Pandemic stressors. Additionally, it is not unreasonable to consider a slight increase of THC use after commercial distribution began with a potential upward trend supersceeding previously observed use rates.

Several different theories may explain this significant decrease in cannabinoid use in pregnancy during the post-legalization period. Changes in substance use reporting, decreased verbal and toxicology screening for substance use by medical providers, impacts of the COVID-19 pandemic on screening priorities and substance use reporting, and ease of access to cannabinoid products may all have impacted the results observed in this study (see Figure 5).

Changes in substance use reporting, including verbal and toxicology screening for cannabinoids during the post-legalization period may be one of the contributing factors as to why there was a decrease in cannabinoid use during this period. Previous studies have shown providers discomfort counseling patients regarding cannabinoid use (49). Additionally, providers are also more likely to use punitive counseling, focusing on the negative social impacts of cannabinoid use (50). This may be due to a lack of general medical knowledge of the effects of cannabinoid use on the developing fetus, which may lead providers to look to other negative impacts of cannabinoids to dissuade patients from use. When cannabinoid products were legalized, many providers may have lost their main punitive counseling point. They could no longer dissuade patients from use solely on the grounds of legality of the drug. This may have discouraged providers from further screening and/or reporting cannabinoid use, leading to the observed results of this study. Additionally, hospital screening policies may change as a result of the legalization status, which could also directly impact the provider having knowledge of cannabinoid use during pregnancy. A recent study in Massachusetts analyzing cannabis use and documentation before and after legalization in the state revealed that despite there being an overall increase in cannabis use related documentation since legalization, only a small portion of medical notes documented actual cannabis use despite the uptake in usage in the state. This suggests a discrepancy between patient reports of cannabis use and electronic medical record documentation of cannabis use (51). Indeed, as the number of individuals identified in Epoch 2 through verbal screen alone was significantly lower than expected, it is very feasible that self-report was not provided and/or utilization of cannabinoids during pregnancy not asked by providers. Thus, the actual use may be overall be higher in the population than what we identified in this review, as the toxicology screening for cannabinoid use during pregnancy decreased following legalization.

Legalization of recreational cannabinoid use in New Mexico took place during the COVID-19 pandemic. The pre-legalization period began January 2019, just before the start of the pandemic, and continued through March 2021. While our logistic regression analysis did not show that COVID-19 alone was a significant contributor to the results of this study, it may have contributed to a complex interaction between multiple factors. A study in California reported increased rates of toxicology confirmed prenatal cannabinoid use during the early phases of the COVID-19 pandemic (from March 2020 to December 2020) (52). This was attributed to COVID-19 related stressors. The initial pre-legalization period included in our study were the earliest stages of the COVID-19 pandemic, at which time lockdown orders were more restrictive, and stressors were potentially higher. Therefore, it is possible the rates of pre-legalization cannabinoid use were higher than normal rates of use during a non-pandemic period, as more individuals used cannabinoid products as a means of stress relief. Our post-legalization period includes the later stages of the COVID-19 pandemic. The decreased rate of cannabinoid use observed in this study may be reflective of a return to a pre-pandemic baseline. In addition, the COVID-19 pandemic led to staffing shortages, and increased stress on healthcare workers (53). These stressors have continued past the COVID-19 pandemic into the present, shifting priorities to screening for viral exposure. Furthermore, an overburdened work force may be less thorough in substance use screening.

As shown in Figure 1, cannabinoid use was decriminalized in April of 2021 and legalized in June of 2021, but retail sales of cannabinoid products as commercial products did not begin until April of 2022. This could explain why we did not observe an increase in cannabinoid use in pregnancy during this period, as individuals may not have had the ease of access to cannabinoid products. It would be interesting to follow the population forward to determine if the utilization increases as the commercial product availability increases and as the world recovers from a pandemic.

As described in other studies, the pregnant population in the PCE group were significantly more likely to use other substances during pregnancy such as tobacco, alcohol, and opioids compared to the control group (see Table 2) (20–22). In evaluating social determinates of health, the pregnant population in the PCE group were more likely to have Medicaid insurance and less likely to have adequate prenatal care (see Table 1) compared to the control group. These findings may have been exacerbated by the COVID-19 pandemic as Epoch 2 showed a significant decrease in prenatal care compared to Epoch 1. It is likely that birthing individuals had higher stress levels due to the stressors of the COVID-19 pandemic which was exacerbated by the higher levels of THC in cannabis products (23, 27–29). No specific markers of maternal stress such as serum cortisol were obtained for this project, however this would be an interesting future direction for additional studies. It is, therefore, not unexpected that the infants in the PCE group had significantly lower birth weights, lengths, head circumferences, 1- and 5-min Apgar scores, and more increased likelihood of birth via Cesarean delivery compared to their counterparts. However, it is difficult to ascertain the extent to which these findings can be attributed to PCE verses the other factors shown to impact infant physical characteristics and health outcomes mentioned above (20–26).

There are several strengths for this study. The data collected by the honest broker through independent chart review of PCE and controls was verified, which decreased errors in data collection and ensured the veracity of the data. As the hospital serves a large geographic region, there is diversity in the participant population. Limitations of the study primarily stem from the nature of a retrospective chart review. The dosage or frequency of cannabinoid use was not typically documented and could not be reliably included in the analysis. The limited window for detecting marijuana metabolites in a urine sample may also have impacted the results, leading to an increase in false negatives. There may be a lead-in period observed during the pre-legalization phase of our study that showed increased THC utilization during this period. As cannabinoid products were decriminalized during the pre-legalization period, individuals may have increased use without the fear of criminal penalties. Additionally, the limitation of the sample size could affect these results. Future analyses completed over time may with a larger sample size may provide additional insights.

While this study looked at the pre-and post-legalization time periods of cannabinoid products in the state of New Mexico, future studies could investigate prenatal cannabinoid use moving forward, as increased commercialization could lead to increased ease of access and higher rates of usage, as would be consistent with other literature. Other studies could look specifically at rates of cannabinoid use in the pre-pandemic pre-legalization period, and the post-pandemic post legalization period to ascertain rates of cannabinoid use without the possible confounding factor of the COVID-19 pandemic.

## Conclusion

Cannabinoid use in pregnancy had a statistically significant decrease during the post-legalization period compared to the pre-legalization period in this study. These findings are contradictory to previous studies which have shown increased rates of cannabinoid use following legalization in both the general and the pregnant population (15–17, 47, 48). Healthcare providers should be aware of these results to underline the importance of continued screening and harm reduction counseling in individuals using cannabinoid products during pregnancy, as the correlation between prenatal cannabinoid exposure and child development continues to be fully characterized. It is critically important to determine the impact legalization of cannabinoid products has on the pregnant population, as this may have lasting impacts for generations to come.

## Data availability statement

The raw data supporting the conclusions of this article will be made available by the authors, without undue reservation.

## Ethics statement

The studies involving humans were approved by University of New Mexico Health Sciences Center Institutional Review Board. The studies were conducted in accordance with the local legislation and institutional requirements. The ethics committee/institutional review board waived the requirement of written informed consent for participation from the participants or the participants’ legal guardians/next of kin because it was a retrospective chart review, thus not requiring consent.

## Author contributions

JT: Data curation, Investigation, Writing – original draft, Writing – review & editing. CM: Data curation, Investigation, Writing – original draft, Writing – review & editing. MA: Data curation, Investigation, Writing – original draft, Writing – review & editing. JG: Formal analysis, Methodology, Writing – review & editing. JM: Conceptualization, Data curation, Formal analysis, Investigation, Methodology, Supervision, Writing – original draft, Writing – review & editing.
